# The Impact of Dementia on Patients with Hip Fracture

**DOI:** 10.15190/d.2024.7

**Published:** 2024-06-30

**Authors:** Andrei Vlad Bradeanu, Iulian Bounegru, Loredana Sabina Pascu, Anamaria Ciubara

**Affiliations:** ^1^Department of Orthopedy and Traumatology, Saint Apostle Andrew Emergency County Clinical Hospital, Galati, Romania; ^2^Faculty of Medicine and Pharmacy, „Dunărea de Jos” University, Galati, Romania; ^3^Competences Centre: Interfaces-Tribocorrosion-Electrochemical Systems, “Dunărea de Jos” University of Galati, Galati, Romania; ^4^Department of Radiology, Saint John Pediatric Clinical Emergency Hospital, Galati, Romania; ^5^Department of Psychiatry, Hospital of Psychiatry “Elisabeta Doamna”, Galati, Romania

**Keywords:** Dementia, hip fracture, hip prosthesis, surgery.

## Abstract

Hip fractures are a serious global health concern with a substantial impact on senior patients' mobility, quality of life, and morbidity. Patients with psychiatric pathology may experience heightened levels of distress, making pain management more challenging. The presence of multiple comorbidities may complicate the therapeutic management of hip fractures. Treatment plans must be carefully tailored to accommodate each individual's unique medical history and current health status. We looked for improving pain evaluation and management in patients with dementia and choosing the best treatment according to age and comorbidities. This study highlights the mortality rate in surgically and non-surgically treated patients and possible correlations with other factors. We conducted a prospective study on 184 patients over 60 years old, with dementia and hip fractures, between 2018 and 2020 in Romania, within the Galati County Clinical Hospital. We applied the Charlson Comorbidity Index, ACE III test, EQ5D5L, and Harris test scores to assess the comorbidities, respectively, pain levels, mobilization in daily life activities, self-care and severity of dementia to exert the optimal treatment for patients with dementia and hip fracture. Our study pointed out that pain was frequently excruciating in non-operated patients compared to those who were operated. Most non-operated patients were immobilized in bed, they required careful and permanent care, while most of the operated patients experienced lower pain levels. While some risk factors of morbidity and mortality, such as comorbidities, severity of dementia, high age, and previous living situations are not preventable, delayed surgery, and general anesthesia risks may be prevented. Despite the treatment, mortality was high both at 6 months and 2 years, with increased survival rate in surgical treated patients. Our study addresses issues such as the importance of mental state evaluation in elderly patients in therapeutic decisions, the surgical intervention and the particularities in pre- and postoperative pain control in patients with dementia, topics that are insufficiently established in the current practical guidelines.

## INTRODUCTION 

Hip fractures represent a major health problem worldwide with significant implications for mobility, quality of life and morbidity in the old patients. The negative impact on the patient's quality of life is determined by the mobility and dependence on relatives. The increase in osteoporosis occurrence has varied in Europe from 246 in Romania in the period from 2005 to 2009 to 677 in the same period in Denmark compared to 100,000 people^1,2^. The total number of hip fractures is expected to double from 2018 to 2050 because of an increase in the number of patients with osteoporosis and dementia. Currently, patients with dementia represent approximately 0,5% of the general population and are 2,7 times more likely to suffer from hip fractures^3,4^. Psychiatric pathology adds complexity and vulnerability to the management of these cases. Through the human aging process, patients' comorbidities worsen and can complicate the therapeutic management as well as the results. The most important complications that can determine the outcome are embolic, infectious and cardiac complications^5^. The risk of death in the first 30 days is 3.8% if the patient was operated in the first 12 hours, thus the surgical intervention after 48 hours increases the risk of mortality at 12 months. About 30-50% of patients with fractures of balance operated with various types of materials become dependent on help from family or qualified staff. Management of hip fractures in patients with dementia differs depending on the country and its financial capabilities^6-8^. It was found that in Norway the risk of mortality after hip fracture in patients who do not have cognitive impairment is 4,8 times higher in men and 2,8 times higher in women^9^. According to European statistics, the incidence rate of hip fractures is higher in Denmark and the UK than in other European developed countries such as Germany^9^. A major cause of hospitalization among older adults is the occurrence of fractures, which represent a public health problem, with emotional, social, and financial impacts^10^. Recurrent fractures occur in 5-10% of patients with this orthopedic pathology, 23% in the first-year post-fracture, and 70% in the first 5 years^11^. The mortality of patients with a hip fracture is high, ranging from 30% to 50% related to the loss of functional independence. Previous studies reported a mortality rate at 1 year ranging between 12% and 33%, 8 times higher compared to the general population^12^. The death rate in patients with hip fractures decreases by 20% if they are operated in the first 48 hours after the traumatic time. Nowadays, the main focus is on the prevention and treatment of osteoporosis. The progressive increase in the risk of death is also related to the patient's current comorbidities. About 75% of patients with hip fractures and dementia encounter a postoperative complication in the first 6 months, and 44% of them suffer multiple complications. 48% of them have minor complications and 52% have major complications. The most common complications include pneumonia, congestive heart failure, and bedsores. Surgical complications may also occur and include incorrect positioning of the implant, wound infections, coxofemoral dislocations due to non-compliance with the instructions of the attending physician. In the vast majority of cases, complications of a surgical nature require a re-intervention, which increases the probability of an in-hospital death^6,12-14^.

## METHODS 

We conducted a prospective study on patients with a mean age of 84,49 years (min. 60 and max. 98 years old), at the Emergency County Clinical Hospital Saint Apostol Andrew, Galati, between January 2018 and December 2020. In this period, 519 patients with hip fractures were admitted to the Emergency Department (ED) in 2018, 520 patients in 2019 and 566 patients in 2020. Of these, only 59 patients in 2018, 60 in 2019 and 65 patients in 2020 associated various types of dementia.

The inclusion criteria in the study were: patients with different types of dementia and acute hip fracture who were treated conservatively or surgically. The exclusion criteria were patients with hip fractures who did not associate dementia and patients with various types of dementia and chronic fractures/diseases or coxarthrosis.

The data was analyzed regarding the frequency of occurrence, using descriptive statistics, correlations and comparisons with the latest studies from the specialized literature. Statistics were performed in Excel and IBM SPSS programs. The patients were selected from the orthopedics ED of our hospital. There were applied several tests for inclusion or exclusion in the studied group. Ace III test was used to detect dementia, EUROQOL and Harris Score were used to evaluate patients after conservative or surgical treatment. The Charlson Comorbidity Index (CCI) was applied to assess the risk of death and included data on age, history of myocardial infarction and tumors, peripheral vascular problems, vascular accident brain, cognitive deficits, lung, gastric, liver and pancreas pathology. Postoperative complications are also important in patients’ prognosis. These tests may be used without restrictions.

## RESULTS

**Figure 1:**
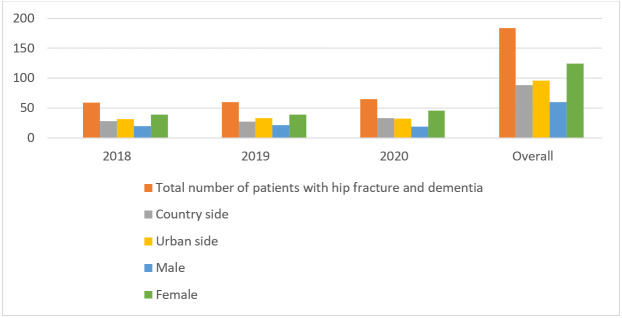
Distribution of patients according to the total number of patients with hip fracture and dementia, the environment and gender. See the increase in the number of patients with both pathologies, with female predominance.

In our study, female predominance was observed with a ratio of 2:1, and higher prevalence in the rural residents (Figure 1). However, the World Bank classification^15^ reported no significant differences between genders, with higher prevalence in low and middle-low countries. Previous studies reported slightly higher incidence in urban areas, but poorer outcomes in the rural residents due to lower health services^16,17^.

Many elderly patients experience severe pain in femoral fractures, due to poor pain assessment or hampered communication with medical staff^18^. Pain is prevalent in patients with dementia, but it is frequently under-assessed and undertreated. About 16% of patients might experience pain at rest, while 57% of patients experience pain during movement^19^. Inadequate pain identification can result in caregiver anxiety, insufficient prescriptions for analgesics, functional deterioration of the patients, and increased risk of falls. Untreated pain raises the risk for primary aggressions, and it is highly correlated with worsening behavioral and psychological symptoms of dementia^20^. Also, this raises the possibility of prescribing unnecessary medications, resulting in polypharmacy and a higher risk of harmful drug responses^21^. Therefore, pain management was a priority in the treatment of our patients, as it improves mobilization and recovery time, and reduces the risk of chronic pain, and hospitalization^22-25^. To assess the pain level, we used the EQ5D5L and Harris Score tests, and there is a good correlation between them of 0.716 in 2018, 0.820 in 2019 and in 2020 it was 0.859, with a mean of 0.798. In the group of patients from 2018, 15,15% of the operated patients did not experience pain after the surgery, while 23,07% non-operated patients were immobilized in bed and experienced severe pain. In 2019, only 8,33% patients among surgically treated patients did not experience pain at all or ignored it, while 19,45% patients experienced severe pain, including supine position. A single non-operated patient 4,17% had no pain, while 33,33%non-operated patients had significant pain levels, including lying in bed. In 2020, only 4,76% operated patients did not experience pain, while 16,67% experienced excruciating pain and were immobilized in bed. Seven 30,43% non-operated patients had mild to moderate pain, while 56,52% patients had permanent pain and were immobilized in bed (Table 1, Table 2, Table 3). In our study, the atrocious pain in non-operated and immobilized patients in the supine position was about 10.1% in 2018, 13.3% in 2019, and 9,3% in 2020. 

*Survival rate in operated vs non-operated patients*

**Figure 2:**
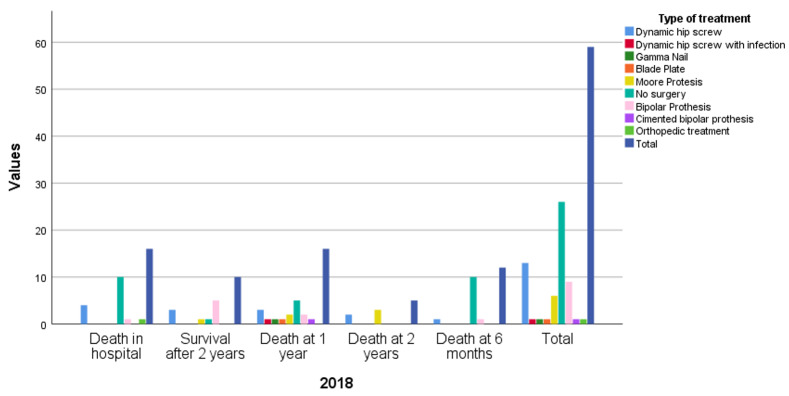
Rate of death in 2018

In 2018, after the hip fracture, 27,11% of patients died during hospitalization, 62,50% of whom were not operated and 37,50% of whom underwent hip surgery as follows: 4 patients had a DHS (Dynamic Hip Screw) system, a single patient with a bipolar prosthesis and one with a reduced dislocation of orthopedic protection. 43 patients were discharged alive and were followed up at 6, 12 and 24 months. At 6 months, 10 non-operated patients, one patient with a bipolar prosthesis and one with the DHS system died, with a high mortality rate of 27,9%. At 1 year follow-up, 5 non-operated patients died, 2 with Moore prosthesis, 3 with DHS system, 2 with a bipolar prosthesis, one with an uncemented bipolar prosthesis, one with DHS with infection and one with Gamma nail. At 2 years follow-up, 5 more patients were deceased, and an increased mortality rate of 65,11%. After 2 years we had 10 survivals, one functionally treated patient, 5 patients with bipolar prosthesis, 3 patients with DHS system and one patient with Moore prosthesis alive (Figure 2). The mortality rate reached 76,74% in the group of followed up patients.

**Table 1:** Pain level assessment using HHS in the group of patients of 2018

			Pain of Hip Harris Score						
Type of treatment			0	10	20	30	40	44	Total
Surgery	EQ5D5L	0	1	-	-	-	-	-	1
		1	-	-	-	-	3	3	6
		2	-	-	-	1	10	2	13
		3	-	-	1	4	-	-	5
		4	-	1	2	-	-	-	3
		5	5	-	-	-	-	-	5
	Total		6	1	3	5	13	5	33
Conservative treatment	EQ5D5L	0	4	-	-	-	-	-	4
		1	-	-	-	-	1	-	1
		2	-	-	1	-	-	-	1
		3	-	-	5	2	-	-	7
		4	-	3	3	-	-	-	6
		5	6	1	-	-	-	-	7
	Total		10	4	9	2	1	0	26
Total	EQ5D5L	0	5	-	-	-	-	-	5
		1	-	-	-	-	4	3	7
		2	-	-	1	1	10	2	14
		3	-	-	6	6	-	-	12
		4	-	4	5	-	-	-	9
		5	11	1	-	-	-	-	12
	Total		16	5	12	7	14	5	59

**Table 2:** Pain level assessment using HHS in the group of patients of 2019

			Pain of Hip Harris Score						
Type of treatment			0	10	20	30	40	44	Total
Surgery	EQ5D5L	1	-	-	-	-	-	3	3
		2	-	-	1	4	6	-	11
		3	-	-	6	1	1	-	8
		4	-	4	2	-	-	-	6
		5	7	-	-	1	-	-	8
	Total		7	4	9	6	7	3	36
Conservative treatment	EQ5D5L	1	-	-	-	-	-	-	0
		2	-	-	-	-	1	-	1
		3	-	-	2	-	-	-	2
		4	-	1	4	5	-	-	10
		5	8	-	2	1	-	-	11
	Total		10	4	9	2	1	0	26
Total	EQ5D5L	1	-	-	-	-	-	3	3
		2	-	-	1	4	7	-	12
		3	-	-	8	1	1	-	10
		4	-	5	6	5	-	-	16
		5	15	-	2	2	-	-	19
	Total		15	5	17	12	8	3	60

**Table 3:** Pain level assessment using HHS in the group of patients of 2020

			Pain of Hip Harris Score						
Type of treatment			0	10	20	30	40	44	Total
Surgery	EQ5D5L	1	-	-	-	-	-	-	0
		2	-	1	1	5	5	2	14
		3	-	1	7	3	9	-	20
		4	-	-	-	-	-	-	0
		5	7	-	-	1	-	-	7
	Total		7	2	8	9	14	2	31
Conservative treatment	EQ5D5L	1	-	-	-	-	-	-	0
		2	-	1	1	1	2	-	5
		3	-	-	2	2	4	-	8
		4	-	2	-	-	1	-	3
		5	6	1	-	-	-	-	7
	Total		6	4	3	3	7	0	23
Total	EQ5D5L	1	-	-	-	-	-	-	0
		2	-	2	2	6	7	2	19
		3	-	1	9	5	13	-	28
		4	-	2	-	-	1	-	3
		5	13	1	-	1	-	-	14
	Total		13	6	11	12	21	2	54

**Figure 3:**
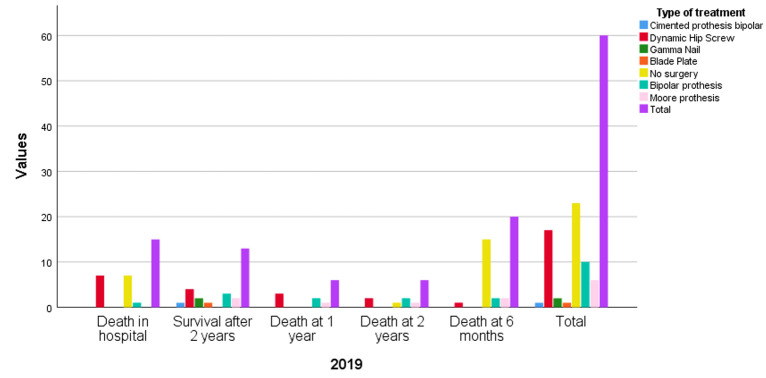
Rate of death in 2019

In 2019, 25% patients died during hospitalization, of which 7 were conservatively treated, one was operated on with bipolar prosthesis and the other 7 patients with DHS systems. There were discharged 45 patients which we followed up at 6, 12 and 24 months, as follows. At 6 months follow-up, there were 44,44% deceased patients - 15 non-operated patients, 2 patients with a bipolar prosthesis, 2 patients with Moore-type prosthesis and one with a DHS system. At 1 year follow-up, out of 6 patients who died, 2 patients had been operated on with bipolar prostheses, 3 patients with DHS systems, a single one with a Moore-type prosthesis and one was conservatively treated, with a mortality rate of 57,77%. At 2 years follow-up, 6 more patients were already deceased, 2 with DHS systems, one patient with bipolar prosthesis, one patient with Moore prosthesis and one patient functionally treated (Figure 3). At the end of our follow-ups, we had 13 survivors and the mortality rate at 2 years was 71,11% among the followed-up group of patients.

**Figure 4:**
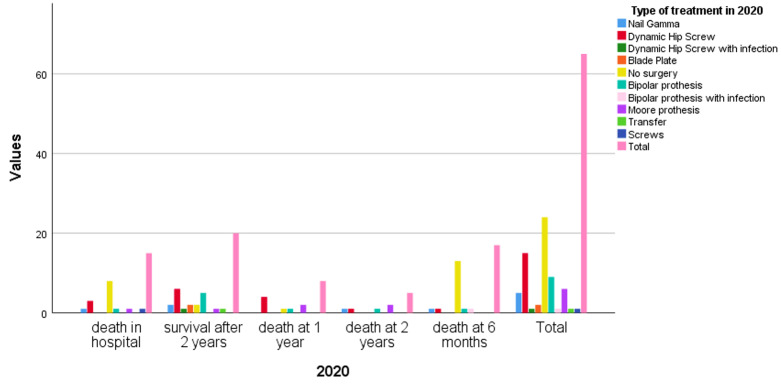
Rate of death in 2020

In 2020, the in-hospital mortality was high, similar to previous years, with 23,07% deceased patients, of which 8 were conservatively treated, 3 had a DHS system, one patient with a bipolar prosthesis, one had a Gamma system, one with Moore-type prosthesis and another patient had osteosynthesis with screws. We followed up the 50 patients discharged alive and noted a mortality rate as follows. At 6 months, 17 patients deceased, with a 34% mortality rate, where 13 patients had been conservatively treated, one patient with a bipolar prosthesis, one with cemented bipolar prosthesis, one with DHS system and another one had a Gamma system. The 1-year follow-up mortality was 38,46%, when 8 more patients deceased, one patient who was not surgically treated, 2 patients with Moore prosthesis, 4 patients with DHS system and another one with bipolar prosthesis. The 2 years mortality reached 46,15%, with 5 more deceased patients, from which, 2 patients with Moore prosthesis and one with bipolar prosthesis, DHS system and Gamma system, respectively (see Figure 4).

The general mortality in our study groups was 27,11% in hospital, 47,45%, 74,57% and 83,05% at 6, 12 and 24 months, in the 2018 group of patients. In 2019, the in-hospital mortality was 25%, and the follow-ups showed a mortality of 58,33%, 68,33%, respectively 78,33%, at 6, 12, and 24 months. The patients from 2020 had an improved mortality rate, with 23,07% in-hospital mortality, 49,23%, and 61,53%, respectively 69,23% mortality rate at 6, 12 and 24 months. The improvement in the survival rate of these patients might be related to the decreased wait of time until the surgery. As stated by Merchán-Galvis et al.^26^ in their study, surgical intervention in the first 24-72 hours after trauma reduces the risk of mortality and complications. In our study, in 2018, the surgery time varied between 2 and 5 days, with a mean of 3,03 days, while in 2019 we had an increased waiting time due to a growing number of hospitalized patients and it ranged between 2 and 6 days, with an average of 3,97 days. During the COVID-19 pandemic, admissions reduced, and was seen an improvement in surgery time, from 2 to 4 days, with a mean of 2,76 years, which correlated with an increased survival rate. However, our mortality remained high compared to previous studies and might be related to the refusal of the patients or the relatives to sign the informed consent regarding the surgical intervention, which led us to use the conservative treatment. Additionally, our study groups generally associated a reduced life expectancy, with a mean age of 84,49 years, while the mean life expectancy in Romania in 2019 was 75.6 years according to the World Health Organization^27^. Our results are consistent with previous results, evidencing the link between dementia and the increased risk of 2-year mortality compared to normal cognition patients, 90-day admission to a permanent care unit risk, and the higher probability of limitations in daily life activities^28,29^. Additionally, increased mortality risk at 1 year was highly correlated with age rather than postoperative complications, except delirium and pneumonia which are considered independent risk factors^12^. 

A multicenter study^30^ on 1491 women reported a higher incidence of hip fractures of 20.8‰, compared to 14.7‰ among demented patients and respectively, the age-matched non-demented patients, but a similar incidence of 20.3‰ in mild cognitive impaired women. The mortality rate was 2 folds higher in patients with dementia at 199.2‰ compared to 89.8‰ in normal cognition patients and higher than 130.9‰ in mild cognitive impaired patients^30^.

Dementia is an independent risk factor in patients with hip fractures. In a Flikweert et al.^12^ study on 479 patients, the mean age was 78.4 years and all patients received a surgical treatment, 62.8% of patients had pertrochanteric fractures operated with various osteosynthesis systems, 34.7% of the patients had femoral neck fractures and were operated with a unipolar or bipolar prosthesis, while only 1.2% were operated with a total hip prosthesis. Their survival rate was 83.6% at 6 months, 82.5% at 12 months and 74.2% at 24 months, but only about 15.4% of patients associated cognitive disorders, while in our study the mean survival rate was significantly lower survival rates 48,09%, 31,70% and 22,96% respectively at 6,12 and 24 months.

Another study with a median age-matched group of patients with our study groups, showed a 46% survival rate at 2 years in patients with dementia and hip fractures, lower than 53,4% in non-demented patients^29^, while our mean survival rate at 2 years was 22,96% (CI 16.95%-30.77%). 

The postoperative evolution of patients with dementia is dependent both on the in-hospital medical act, and also on the postoperative rehabilitation to restore the mobility before the fracture as much as possible. Medical recovery is particularly important in the case of patients who suffered a hip fracture and underwent surgery, but especially if they also have dementia. Each dementia patient may have specific needs. A recovery plan should be tailored to the patient's level of functioning and cognitive ability. It is important to set realistic goals and monitor progress regularly. Due to the cognitive deficits of the patients, careful supervision is necessary throughout the performance of the recovery exercises to avoid the occurrence of new traumas. A recent study^31^ showed that 82.2% of patients with dementia and operated hip fracture will benefit from a recovery in a special center and only 57.6% of patients with operated hip fracture but without dementia will opt for physical therapy. Another study^28^ reported higher admissions to a rehabilitation center within 2 weeks in non-demented patients 70,1% compared to 67,3% in demented patients. Since the health infrastructure is under development in our country, and the geographical area does not yet have a rehabilitation hospital, the patients in our study are forced to follow a rehabilitation program in private clinics, and a very small percentage of them may afford to follow a program of full rehabilitation.

## DISCUSSIONS

Pain intensity can be considered to have clinical value, even if it can occasionally be challenging to show meaningful correlations between pain intensity and recovery. Variations in research outcomes might result from a multitude of factors, including study scope, questionnaire design, pain treatment techniques. Non-operated patients had unbearable pain and were immobilized in bed in most cases, on the other hand, the majority of operated patients had minor or even transient pain, however, less than 50% might restore to their pre-fracture mobility and daily life activities. 

The frontline medical staff in charge of managing pain are nurses, and they must have correct pain assessment skills, particularly in a patient with significant cognitive impairment who is unable to talk. They must shift from utilizing self-report as a standard method of assessment to relying on behavioral pain displays using an observational pain measure. The brain regions implicated in coding perception and language are damaged in severe demented patients, making pain observational evaluation difficult and dementia-related memory loss can compromise a person's capacity to exhibit pain behaviors, or muscle memory^31,32^. Nurses lack of confidence in assessing pain in demented patients may impact management, as they rely on nonverbal cues, family, and previous hospital notes to confirm pain assessments. Additionally, pain assessment tools often are not routinely used in daily practice and the medical staff rely on past experiences, such as changes in behavior, facial expressions, and analgesic prescriptions, to assess pain^25,33^. The staff may be more inclined to conduct routine pain assessments and document the results if the pain is evaluated on a group level and is given a mode and maximum value. Currently, there are numerous tools, but it is no gold standard tool for pain assessment in patients with severe dementia. We expect automatic tools for facial recognition of pain and artificial intelligence to be implemented in future clinical practice. ePAT is the first automated tool for pain facial analysis that seems to have promising results, with high sensitivity (96.1%) and specificity (91.4%)^34,35^. According to a study published in the Journal of Pain and Symptom Management, realized on 97 patients with hip fractures, among which 38 patients associated with dementia, of which 40% had severe postoperative pain. The 59 patients who have preserved cognitive function, mainly present moderate to severe pain in the first 3 days after the intervention. The use of anti-inflammatories was 18.9% and 23.2% respectively in patients with dementia in the first year after the fracture, but the use of opioids increased in both groups of patients^36,37^. Another observational study on 32379 patients, reported shorter opioid administration than in patients without dementia, but no statistical significance was seen, suggesting that postoperative analgesia in demented patients may be insufficient in many cases^38^.

Additionally, nurses must regularly gather information on how a patient's degree of pain impacts their ability to walk, move about, maintain personal hygiene, sleep, and eat. Upon post-operative patient discharge, pain management and healing are critical considerations. Inquiring into the patients' experiences with pain is crucial for determining the most appropriate course of action or therapy (i.e. the early postoperative recovery)^24,39^. 

In the medical field, there is still a wide uncertainty in pain management in patients with dementia. Some previous studies reported lower doses of oral morphine equivalents during the first 48-hour postoperative, that increased on the 3^rd^ day, similar to non-demented patients or administered when required^37,40^. Other studies reported higher doses of opioids in demented patients in the first 72 hours than the general population^32^. 

A few studies reported the use of nerve blocks before hip surgery as a promising alternative for opioid administrations, as it is already known that is difficult to adjust and side effects are not neglectable (associated with sedation and mental function impairment)^25,37,40^. There were described two different techniques – in the Unneby et al.^41^ study, the femoral nerve block was performed with levobupivacaine in the first place and a secondary nerve block was used when the surgery was postponed. Garlich et al^42^ used bupivacaine as a fascia iliac block and a continuous nerve catheter until the next morning, before surgery. Both techniques were correlated with postoperative lower pain scores, lower doses of administered opioids, and subsequent less opioid-rated side effects^25^. Moreover, continuous nerve blocks are useful in prolonged analgesia, reducing and sometimes avoiding opioid use^43^.

International Guidelines recommend nerve blocks use as a good alternative to analgesia, but without postponing the surgery^43-45^. Meanwhile, another study shows that patients with hip fractures with associated dementia had lower chances of receiving nerve blocks^46^. 

Patients after hip fractures with moderate dementia may exhibit improvements in function and mobility, as well as a reduction in fall risk, that are comparable to those made by those without dementia^28,47^. 

Our results are consistent with previous studies which found that the admission cognitive status is highly correlated with motor functional independence measures independent from delayed mobilization^28,29^. According to previous studies, demented patients had a decreased tendency to early mobilization and a lower survival rate^48,49^. Nevertheless, patients with moderate dementia improved more after the hip surgery in terms of independence at 3 months than those with or without severe dementia, according to a subgroup analysis of a study of rehabilitation in patients with hip fractures^50^. Otherwise, physiotherapists complained about the difficulty in following the standard protocols in the rehabilitation of demented patients, which are inappropriate or unachievable by those patients. Therefore, postoperative recovery in a qualified center is necessary for improving mobility, self-care capacity, communication and social activity. It also increases the survival rate over a longer period^51,52^. Early mobilization was correlated with reduced hospitalization, and improved survival rates at short and medium term^49,53^.

*Limitations of the study*

The inability to operate on all patients with hip fractures and dementia due to the indecision and refusal of the relatives, despite the recommendations of the medical professionals. Postoperative care in specialized recovery centers progressively increases the survival rate. Last but not least, the COVID pandemic has created barriers between health personnel and patients.

## CONCLUSION 

This article contributes to the understanding of the complex interplay between hip fractures, dementia, and postoperative care, offering important implications for improving clinical practices and patient outcomes in the medical field. Early surgical intervention and mobilization may enhance pain control patients’ outcomes, and quality of life and were correlated with increased life expectancy. Pain assessment tools should be encouraged for routine use in medical practice. Implementation of automated tools for pain assessment (like ePAT) may improve care for dementia patients. Tailored postoperative protocols dedicated to demented patients may emphasize pain control.
